# Waist Circumference and Abdominal Obesity among Older Adults: Patterns, Prevalence and Trends

**DOI:** 10.1371/journal.pone.0048528

**Published:** 2012-10-31

**Authors:** Denise Howel

**Affiliations:** Institute of Health & Society, Newcastle University, Newcastle upon Tyne, United Kingdom; Dana-Farber Cancer Institute, United States of America

## Abstract

**Objectives:**

To describe the patterns and trends in waist circumference and abdominal obesity for those aged 70–89 contrasting the standard and new age-related cut-points, and to investigate how they vary with time, age and educational level.

**Methods:**

The subjects were 7129 men and 9244 women aged 70–89 years who participated in the Health Survey for England during 1993–2010. The outcome measures were the percentiles of waist circumference and standard and new indicators of abdominal obesity based on waist circumference. Binomial and quantile regression were used to investigate the relationship with key explanatory variables.

**Results:**

The distribution of waist circumference among community-dwelling older adults in England has shifted upwards since 1993 (an increase in median of 4.5 cm in men and 5.1 cm in women). The prevalence of abdominal obesity has increased, while those in the low-risk group have decreased. Abdominal obesity was higher in those aged 70–79 compared to 80–89, and in those who left education earlier. The prevalence of abdominal obesity varies considerably with new and standard cut-points, which makes it impractical to use the new ones on a population that includes subjects across the adult age range.

**Conclusions:**

Obesity is increasing among the elderly, but more work is needed on devising age-appropriate indicators of high risk based on waist circumference.

## Introduction

The rising global trends in generalized obesity, defined using body mass index (BMI), have been well described [1], [2]. It has been argued that waist circumference (WC) is as good, or even better, as a measure of excess adiposity than BMI [3]–[7], particularly among older adults, because of the age-dependent height decrease [8], [9]. Waist circumference can be considered as a continuous or binary variable, using cut-points to indicate high risk values (abdominal obesity and overweight). However, questions have been raised about whether the well-established cut points for adult waist circumference should be age-specific [10]–[13]. It has been suggested that WC cut-points should be shifted upwards in older adults, and new values have been suggested for adults aged 70 and over [14]. The aim of this study was to describe the patterns and trends in waist circumference and abdominal obesity and overweight in England for those aged 70–89 (using both the standard and new cut-points) and investigate how they vary with time, age and educational level.

## Methods

The Health Survey for England (HSE) is a series of annual cross-sectional surveys. The analyses in this paper come from the core population samples from the HSE between 1993 and 2010: however waist circumference was not collected in the core samples in 1995–1996, 1999–2000 and 2004. Geographically representative private households were identified using multi-stage sampling and all adults therein invited for interview. A new sample was invited every year. Trained interviewers collected socio-demographic information at the homes of participants. WC was defined as the midpoint between the lower rib and upper margin of the iliac crest, measured by a nurse using a tape with an insertion buckle at one end. The measurement was taken twice and recorded to the nearest even millimetre. The response rates varied across each survey but around 70% agreed to an interview, and WC was available on around 90% of interviewees. Further details of the survey methodology and results are available in published reports and online [15], [16]. The datasets were downloaded from the UK Data Archive.

The outcome measures used were the percentiles of waist circumference and indicators of abdominal obesity and overweight. The standard cutoff values for abdominal obesity and overweight in Europid adults are WC≥102 cm and ≥94 cm in men, and ≥88 cm and ≥80 cm in women [17]–[19] and were originally developed to reflect those for obesity based on BMI. Note that the overweight categories don’t include those who are obese i.e. 94–101 cm in men and 80–87 cm in women. Recent work has considered optimal cut-points for abdominal obesity, and recommended that for those aged 70 years and older the cutoffs should be 100–106 cm in men and 99 cm in women [14]. To illustrate the maximum change to the estimate of obesity prevalence, the cut-off for men has been set at 106 cm in the following analyses. The analysis was restricted to the age range 70–89 years. The lower limit was to allow use of the new set of cut-off values for large waist circumference developed in adults aged 70 years and older [14]. The upper limit was set at 89 years, since only a very small proportion of the HSE sample were aged 90 years or over. A total of 7129 men and 9244 women were included in the analyses (43% of the sample were male, and 75% of males and 70% of females were aged 70–79 years).

The prevalence of abdominal obesity (standard and new definitions) was plotted against survey year using lowess smoothing to illustrate any trend. The relationships between the prevalence of abdominal obesity (standard and new definitions) with age, education and survey year was fitted by generalized linear models with binomial errors and an identity link function. Education was included as a proxy for socioeconomic position and categorized as those who left school at <16 or ≥16 years. Age was split into two 10-year age bands: 70–79 and 80–89 years. Four separate models were fitted for the two outcome measures in both men and women, with the explanatory variables being age-band, survey year, and educational group. Interaction terms between the three factors would only be included if they were significant at the 1% significant level.

The shift in the distribution of waist circumference between 1993/4 and 2009/10 was illustrated by a smoothed kernel density plot of the distributions. Simultaneous quantile regression was used to fit a model of the 15^th^, 50^th^ and 85% percentiles of the WC distribution (separately for men and women) based on survey period, education and age-band. Hypothesis tests were carried out as to whether regression coefficients differed across the percentiles. All statistical analyses were done using the statistical package STATA version 12.

## Results

**Figure 1:**
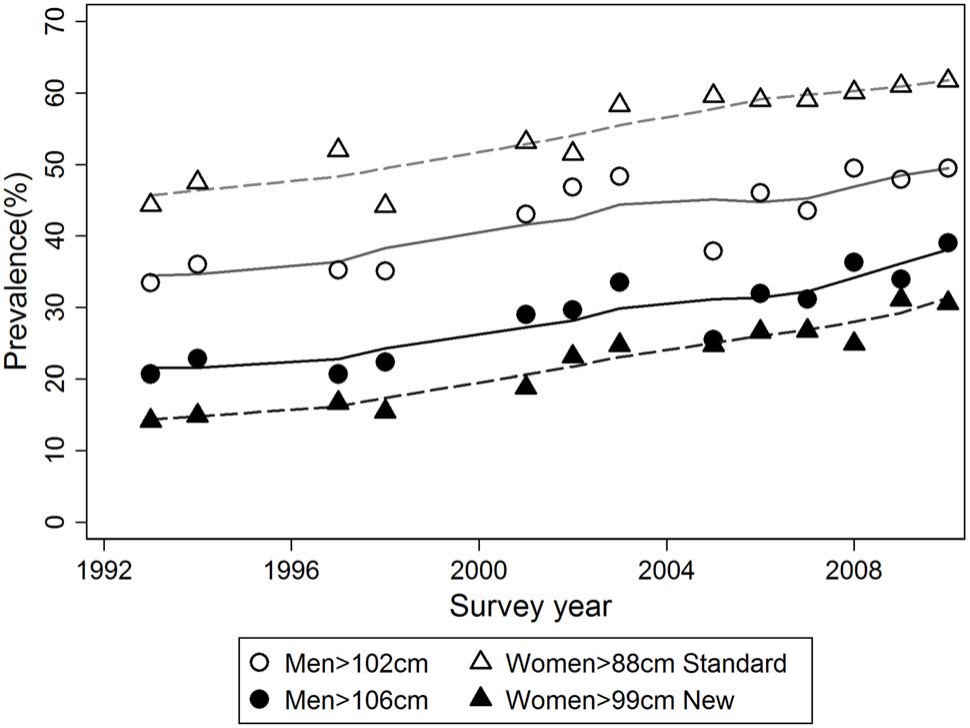
Prevalence of high waist circumference (WC) using new and standard cut-offs in English adults aged 70–89 years during 1993–2010.

Between 1993/4 and 2009/10, the prevalence of abdominal obesity, using the standard and new definitions, rose in both men and women aged 70–89 years in England. The distribution of men in the Low risk/Abdominal overweight/Abdominal obese categories (standard definition) changed from 34.5%/30.7%/34.8% in 1993/4 to 21.0%/30.1%/48.9% 2009/10, whereas in women it changed from 25.7%/28.5%/45.8% in 1993/4 to 16.5%/22.4%/61.1% in 2009/10. In general, abdominal obesity increased, the proportion in the normal waist circumference band decreased, and the proportion in the abdominal overweight band was stable. Abdominal obesity was more common in women than men of this age group throughout the study period, using the standard gender-specific cutoffs. The trend across the whole time period is illustrated in Figure 1, which shows the results both for the standard and new definitions of abdominal overweight and obesity in older people. It can be seen that the trend in both obesity outcomes is approximately linear over the whole period. It is also clear, when comparing the prevalence calculated using new and standard cut-offs, that the prevalence of abdominal obesity is markedly lower, and abdominal obesity is more common in men than women using the new cut-offs.

Table 1 shows key results of the binomial regression analyses modeling the prevalence of abdominal obesity in men and women. A linear trend in survey year was found to be the best fit in all models of obesity, and age group and educational group were significantly associated with the prevalence. In these models the estimated abdominal obesity prevalence rose by about 1% per year, was higher in the younger age group by around 5% and higher in those who left education earlier by around 4% depending on the outcome. No interaction terms with survey year were a significant improvement to the model (P>0.01), which therefore showed little evidence of widening inequalities with educational group or age-group over time.

**Table 1:** Binomial regression parameters in the four models of prevalence (%) of abdominal obesity in English men and women aged 70–89.

	Regression parameters (95%CI)		
Dependent variable	Survey year	Age group70–79 vs 80–89 yrs	Left education<16 vs ≥16 yrs
MEN (n = 7129)			
Obesity (standard cutoff) 1	1.0(0.8 to 1.2)	6.2 (3.6 to 8.7)	4.6 (2.0 to 7.1)
Obesity (new cutoff) 2	1.0 (0.8 to 1.2)	4.4 (2.1 to 6.7)	4.4 (2.2 to 6.6)
WOMEN (n = 9244)			
Obesity (standard cutoff) 1	1.1 (0.9 to 1.3)	4.2 (2.0 to 6.4)	4.8 (2.6 to 7.1)
Obesity (new cutoff) 2	1.0 (0.8 to 1.1)	4.6 (2.9 to 6.2)	3.3 (1.5 to 5.0)

1 Standard cut-off Abdominal Obesity - WC >102 cm (men) and >88 cm (women).

2 New cut-off Abdominal Obesity - WC >106 cm (men) and >99 cm (women).

**Figure 2:**
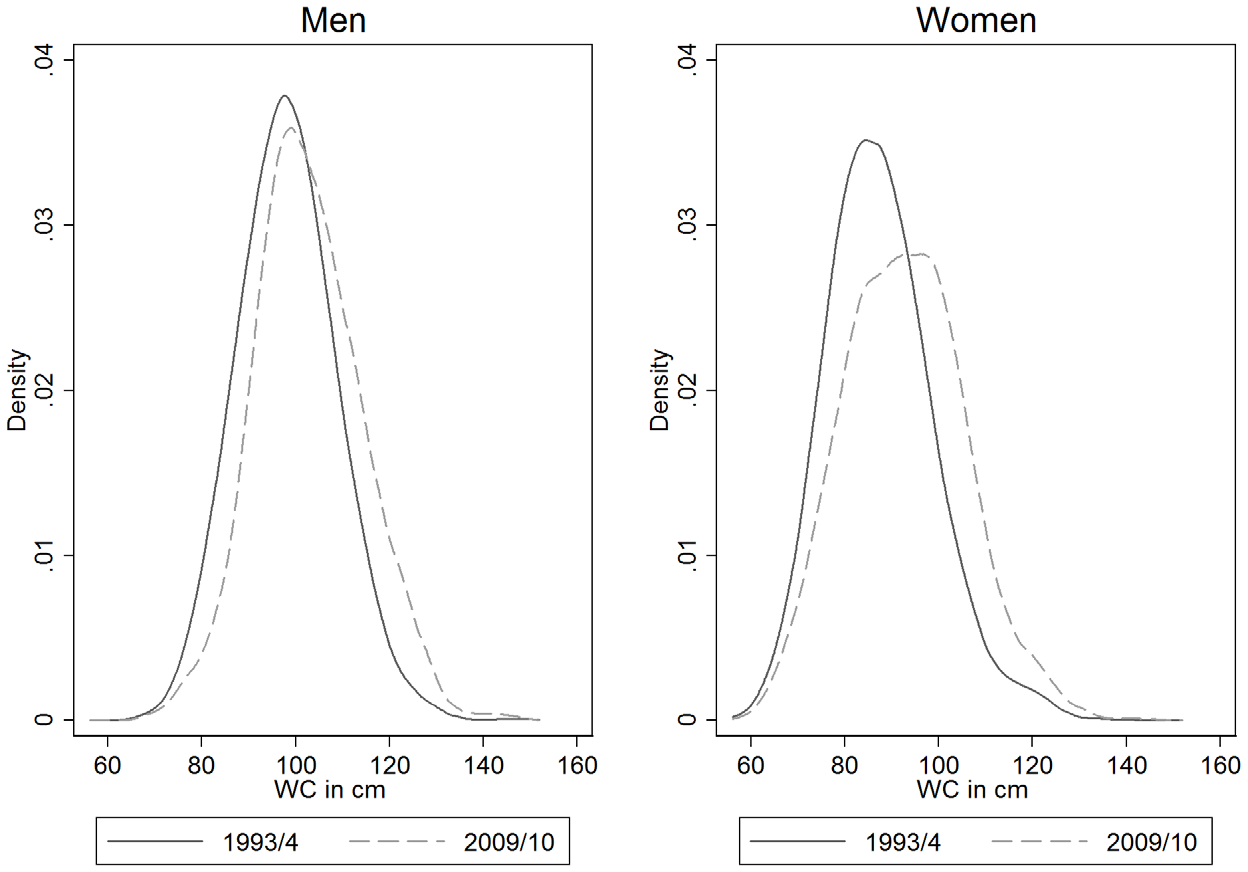
Changes in distribution of waist circumference between 1993–4 and 2009–10 in English adults aged 70–89.

The median waist circumference in men rose from 98.2 cm in 1993/4 to 102.4 cm in 2008/9, while the change in women over the same period was from 87.5 cm to 91.6 cm. The distributions of waist circumference measurements are contrasted for 1993–4 and 2009/102 n Figure 2. It can be seen that the distribution has shifted upwards over time, and appears to have changed more at the upper end of the distribution. This was confirmed by the results of the quantile regression shown in Table 2. For both men and women, all three percentiles increased significantly over time and there was a significant difference between the regression coefficients for survey year across the 15^th^, 50^th^ and 85^th^ percentiles (P = 0.002 for men and P<0.0001 for women), such that the coefficients showed a positive gradient with percentiles, suggesting that the gains in waist circumference over time have been greater at the upper end of the distribution. All three percentiles were significantly higher in the younger age group for men and women, but there was no significant difference in coefficients across the percentiles (P = 0.94 for men and 0.16 for women). In men, there was no significant difference in the 15^th^ percentile between the educational subgroups, but the 50^th^ and 85^th^ percentiles were significantly higher in those who left education earlier. In women, all three percentiles were significantly higher in those who left education earlier, but there was no significant difference in coefficients across the percentiles (P = 0.13).

**Table 2:** Quantile regression coefficients of waist circumference distribution(cm) predicted from survey year, age group and educational group.

MEN		Percentiles						
Variable		15%		50%		85%		P-value^1^
Year		0.27 (0.21 to 0.34)		0.28 (0.22 to 0.33)		0.41 (0.33 to 0.49)		0.002
Age group^2^	70–79	1.6 (0.8 to 2.5)		1.9 (1.1 to 2.8)		1.6 (0.5 to 2.7)		0.94
Left education^3^	<16 yr	0.1 (–0.6 to 0.8)		1.3 (0.6 to 1.9)		1.9 (1.0 to 2.8)		0.0007
Constant^4^		86.0 (85.0 to 87.0)		95.2 (94.2 to 96.2)		106.9 (105.9 to 107.9)		
**WOMEN**		**Percentiles**						
Variable		15%		50%		85%		P-value^1^
Year		0.22 (0.15 to 0.29)		0.36 (0.30 to 0.41)		0.47 (0.40 to 0.53)		<0.0001
Age group^2^	70–79	1.3 (0.6 to 2.0)	1.4 (0.7 to 2.1)		2.1 (1.2 to 2.9)		0.16	
Left education^3^	<16 yr	0.8 (0.1 to 1.6)	1.6 (0.8 to 2.4)		1.7 (0.7 to 2.6)		0.13	
Constant^4^		74.5 (73.3 to 75.6)	84.0 (83.0 to 85.0)		95.2 (94.1 to 96.3)			

1 Hypothesis of equal coefficients for a variable in the regression equations for the 15%, 50% and 85% percentiles.

2 Compared to age 80–89.

3 Compared to left education ≥16 years.

4 Corresponds to predicted percentile for year = 1993, age-group = 70–79 & left education ≥16 years.

## Discussion

An upward linear trend was seen in the prevalence of abdominal obesity, in both community-dwelling older men and women, between 1993 and 2010, and conversely, an almost matching decrease in those with a low-risk waist circumference. A similar rise in abdominal obesity was also seen in adults aged 18–67 in England over the same period, with some evidence that the rates of increase were slowing down [20]: however there was no evidence of a slowdown in the 70–89 age group. To put this trend in older adults in context, other factors associated with obesity have also changed between 1993 and 2010: the average age in the 70–89 age-band in the survey has increased slightly from 76.2 to 77.0 years, and the proportion that left school before the age of 16 has decreased from 74.8% to 64.1%. However, after adjusting for age and educational category, a linear trend in abdominal obesity and overweight was the best fit to the data, and there was little evidence that the time trend varied between educational or age-groups.

There are relatively few studies which have reported trends in abdominal obesity in older people. An analysis of National Health and Nutrition Examination Survey (NHANES) data in the United States reported a significant trend in abdominal obesity from 1999/2000 to 2007/8 in men aged over 60 years, but the rise amongst older women was not significant [21]. Earlier comparisons of NHANES data in those aged over 70 years between 1988/94 and 1999/2000 also found a significant trend upwards in men, but not in women [3]. This was a similar age group to those in this study and showed that abdominal obesity was more common in the US than England over the comparable time period. Elsewhere, surveys in Korea found that there had been a significant increase in abdominal obesity in both men and women aged over 60 between 1998 and 2007 [22].

The abdominal obesity prevalence estimates are much lower using the new cut-points, which obviously has implications for the interpretation of the values. Another difference between the new and standard cutoffs is that the prevalence of abdominal obesity in those aged 70–89 is higher in men using the new cutoffs, but higher in women using the standard cut-points : this latter pattern mirrors the finding that abdominal obesity is more common in women aged 18–67 in England [20]. This will be partly explained by the fact that the cutoffs have been raised beyond the standard values by 4 cm for men (and this was the maximum suggested) and 11 cm in women [14]. The standard cutoffs were originally devised to reflect the relationship between BMI and WC and waist-hip ratio. Higher cut-points for WC have been suggested previously to retain this relationship between BMI and WC in older people [12], but there has been discussion over whether the usual BMI and related cutoffs are appropriate for older adults given the age-dependent decline in height [9]. The new ones used here have been devised to detect a high risk of a number of health outcomes (mobility limitations, pain, incontinence, knee osteoarthritis, diabetes, cardiovascular disease) [14] which are important negative outcomes in the elderly. Given the difference in the target outcomes for the new and standard cut-points, it is not surprising that there is a large difference in the cut-points chosen. However, at present, this does make it impractical to use the new cut-points on a population that includes subjects with ages either side of 70 years [10]–[12], [23] If there is a justification for raised cut-points for older adults, they would be more useful if they incorporated a gradual change with age, but little work has been done so far on devising age-appropriate cut-off values across the adult age range based on the most relevant health risks. It can be seen that estimates of the prevalence of high risk waist circumference are highly sensitive to the cut-off chosen, and it is important that more work is done to find an agreed way of monitoring obesity using simple anthropometric measures that are relevant to adults of all ages.

As expected, the prevalence of abdominal obesity was lower in the age-group 80–89 compared to 70–79 years. There isn’t much published data that distinguishes between the prevalence in these two age-bands, but this pattern was also seen in two studies in Spain [24], [25]. However, this pattern needs to be interpreted with care. 72% of the sample were aged 70–79 years as opposed to 28% aged 80–89, and it is not clear what form any survival bias may take. It has been argued that the obesity-mortality relationship flattens with age because those susceptible to the effects of adiposity have died [9]. However, it has also been noted that those with highest risk of mortality are in the lower ranges of BMI, so selective survival could lengthen the survival of older people who are obese [25].

There is substantial research on the links between measures of obesity and measures of socio-economic position(SEP). A international review of studies on SEP and obesity found that most studies in more developed countries reported associations between lower SEP and obesity in women, though the associations were more likely to be non-significant in men [26]. One measure of SEP is educational level, and a recent review of educational inequalities and obesity and overweight in European countries echoed the pattern seen of SEP measures in general with obesity [27]. When specifically considering abdominal obesity, surveys in Britain have also found a social gradient in its prevalence among both men and women, though the evidence for this relationship in men was not always very strong [28]–[31]. However this previous research was often based on samples where the average age was much lower than was seen in this study. There has been speculation as to how far social disparities persist in the oldest section of the population [32]. However, in this study we found that the prevalence of abdominal obesity was significantly higher in those who had left school before the age of 16 years.

When waist measurements were considered as continuous variables, it was seen that the distribution has shifted upwards between 1993/4 and 2009/10 and but that changes were greater at the upper end of the distribution: the quantile regression results confirmed this. All three percentiles (15^th^, 50^th^ and 85^th^) were lower in those aged 80–89 compared to 70–79 years in men and women, but there was no indication that losses occurred at any particular part of the distribution. In men, there was some evidence that the educational inequalities were greater at the upper end of the distribution, but in women, all three percentiles were significantly higher in those who left education earlier, with no significant difference in coefficients across the percentiles. An analysis of data on adults aged 18–64 from the Health Survey for England between 1993/4 and 2002/3 also found that the distribution of waist circumference had shifted upwards over the period and predominantly at the upper end of the distribution [33]. However they did not find that changes in percentiles were associated with educational level. An analysis of data from the US between 1960 and 2000 found that the distribution of waist circumference had shifted to higher values and there was a significant upward trend in mean WC in all age groups, including those aged 70–79 years [34].

An advantage of the HSE data is that it is from nationally representative surveys of residents in private households, where waist circumference was measured by nurses. Unfortunately WC was not collected in the core sample every year, but sufficiently often to give good estimates of any trends over time. Given that this study was concerned with those aged over 70 years, it is possible that the non-inclusion of residents of institutions may have led to a less representative sample in this age group: however there is no evidence either way. Those in some subgroups may be less likely to respond. The methodological reports of the HSE compared the age and sex distribution of HSE participants with that from the national Census, and have found that women and older people are slightly over-represented. However the Census also included the subgroup living in institutions, which is not included in the HSE, and this makes it difficult to estimate the extent of any bias. The HSE introduced weighting for non-response in 2003, but these weights were only available for six of the years included in this study.

The distribution of waist circumference among older adults in England has shifted upwards since 1993 and, correspondingly, the prevalence of abdominal obesity has increased. However, although considerable efforts have been devoted to population strategies to reduce obesity in general, less encouragement has been given to weight management in older people. There is relatively little evidence as to whether the advantages of voluntary weight loss outweigh the risks of loss of muscle mass and bone density [9], [35]. Nevertheless, obesity has been shown to be associated with several disorders in old age such as metabolic syndrome, cardiovascular disease, osteoarthritis and lack of mobility [4], [36]. This is likely to be reflected in increased medical and social needs, so weight-loss therapy that minimizes muscle and bone loss has been recommended for older people who are obese [36]. Unless progress is made in reducing obesity in the elderly, this will result in an increasing burden on health-care services.
